# Public perceptions of AI in healthcare: a large-scale BERTopic and sentiment analysis of Reddit discussions

**DOI:** 10.3389/fpubh.2026.1839898

**Published:** 2026-06-02

**Authors:** Zaiyu Tang, Wenbao Ma, Zhipeng Bai, Jiahe Liang, Yandong Xie

**Affiliations:** 1 Department of Philosophy, Xi’an Jiaotong University, Xi’an, Shaanxi, China; 2 Department of Marxism, Party School of the CPC Shanxi Provincial Committee (Shanxi Academy of Governance), Taiyuan, Shanxi, China; 3 3D Printing Research Center, Department of Ultrasound Medicine, The Second Affiliated Hospital of Air Force Medical University, Xi’an, Shaanxi, China; 4 Department of Gastroenterology, The Second Affiliated Hospital of Air Force Medical University, Xi’an, Shaanxi, China

**Keywords:** artificial intelligence, healthcare, public perception, Reddit, sentiment analysis, social media, topic modeling

## Abstract

**Introduction:**

Public perception plays an important role in the responsible implementation of artificial intelligence (AI) in healthcare because trust, perceived risk, and expectations regarding human–AI collaboration may influence the acceptance of AI-assisted medical services. This study aimed to examine public discourse and sentiment regarding AI in healthcare using large-scale Reddit discussions.

**Methods:**

We conducted a retrospective content analysis of 36,555 Reddit posts and comments published between March 1, 2020, and March 31, 2025. Reddit was used as a source of large-scale, spontaneous, user-generated discussions. BERTopic modeling was applied to identify latent discussion topics. Topics were interpreted based on semantic similarity, representative keywords, and representative paraphrased posts, and were subsequently grouped into thematic domains. Sentiment analysis and temporal trend analysis were also performed.

**Results:**

Fourteen discussion topics were identified across six thematic domains: human-centered healthcare, auxiliary medical services, AI platforms and tools, cultural perceptions, food and health safety, and medical regulation. Overall sentiment distribution was 41.4% positive, 23.8% neutral, and 35.1% negative, indicating a generally positive orientation while also revealing substantial public concern. Negative sentiments were primarily associated with technological maturity, commercialization, privacy and safety risks, and the potential displacement of physicians. Temporal analysis demonstrated changes in sentiment distribution over time, particularly following the widespread public diffusion of generative AI tools.

**Discussion:**

The findings suggest that public attitudes toward medical AI are simultaneously optimistic and cautious. Concerns regarding governance, safety, commercialization, and workforce implications remain prominent in online discussions. These results highlight the importance of transparent communication, clearer regulatory governance, and careful workforce planning to support the responsible integration of AI into healthcare systems.

## 1 Introduction

Artificial intelligence (AI) is increasingly being integrated into healthcare, where it is expected to support diagnosis, clinical decision-making, administrative work, patient communication, and medical robotics (1–3). These applications illustrate the practical promise of medical AI, but they also raise questions about reliability, privacy, accountability, professional roles, and the preservation of human-centered care (4–10). Therefore, understanding how people discuss and evaluate medical AI is important for its ethical and clinically responsible implementation.

Although AI is expected to reshape future clinical practice (11), its application potential and associated challenges continue to draw extensive attention from both academia and society (7). At the same time, its clinical use raises ethical, regulatory, and human-centered challenges. Developers often prioritize analytical capabilities, accuracy, and data processing speed while relatively neglecting the integration of human perspectives (12). This narrow technological focus limits the potential for clinical translation. Additionally, public concerns regarding the reliability, privacy security, and accountability of medical AI cannot be ignored (9, 13, 14). Therefore, gaining a deep understanding of public attitudes is essential because acceptance of medical AI depends not only on technical performance, but also on perceived usefulness, perceived risk, trust, and the protection of patient-centered values.

Currently, only a few studies have explored public attitudes toward medical AI by collecting user discourse on social media regarding medical AI or related technical fields (15–20). However, these works either lack systematic data analysis or fail to disclose statistical data and methodologies. To address this gap, this study analyzes Reddit discussions through an integrated framework that combines topic modeling, sentiment analysis, and temporal trend analysis to examine online discussions and emotional attributes surrounding medical AI. This design allows the study to move from general observations of online discourse to a more structured analysis of what users discuss, how they evaluate it, and how these evaluations change over time.

We primarily focus on the following three research questions:

1 What are the predominant topics in public discussions regarding medical AI?
2 What are the prevailing sentiments among the public surrounding medical AI?
3 How did public attitudes toward medical AI evolve over time between March 1, 2020, and March 31, 2025?

The primary contributions of this paper are as follows:

1 We utilize real-time social media data to rapidly identify trends and patterns in the public domain (21). To the best of our knowledge, this study is the first to provide insights into AI in healthcare from the extensive and diverse perspectives of Reddit users while making all relevant data available.
2 We integrate BERTopic-based topic modeling with large language model-based sentiment analysis and trend analysis, allowing us to examine topics, sentiment, and temporal patterns from complementary perspectives. All data disclosable under privacy protocols, along with data analysis and statistical methods, are accessible at https://doi.org/10.5281/zenodo.18811076.
3 We further identify common themes and attitudes expressed in social media discussions about healthcare AI, thereby offering diverse societal perspectives on relevant public opinions and emotions. These insights may help practitioners and policymakers better understand public concerns about the responsible implementation of medical AI. The following literature review therefore first summarizes existing evidence on attitudes toward medical AI and then explains why social media discourse can complement survey- and interview-based approaches.

## 2 Literature review

In the existing literature, most studies employ questionnaires (22–31), interviews (28, 32, 33), or surveys (32, 34, 35) to investigate public attitudes toward medical AI. These methods are widely used to examine public attitudes across different fields (36). Research indicates that subjects from different professional backgrounds exhibit significant attitudinal variations regarding healthcare AI technologies, generally presenting a complex stance characterized by cautious optimism or conditional acceptance.

Healthcare professionals generally maintain a positive outlook (26, 30–33). For instance, Wang et al. (30) found that nurses are open to AI applications in nursing, believing they can alleviate administrative burdens and improve patient monitoring. A nationwide survey by Xia et al. (31) targeting Chinese radiologists and trainees also showed that respondents generally welcome GPT-like technologies, recognizing their potential in report generation and education. Findings by Al Saad et al. (22) suggest that medical students broadly support the application of AI in the medical field. However, this support is often accompanied by conditions and reservations (24). Dean et al. (24) found that while frontline physicians and physician assistants in Kansas, USA, acknowledge the value of AI in enhancing efficiency, they expect AI tools to be user-friendly and seamlessly integrated into existing workflows; they also expressed concerns regarding increased cognitive load or the risk of de-skilling in clinical decision-making. Furthermore, professional attitudes vary based on individual circumstances and occupational environments. Reffien et al. (28) identified significant differences in attitudes toward AI use between doctors in clinical and non-clinical departments, with non-clinical physicians being more positive, highlighting the influence of daily work exposure on perception.

While patients and the general public maintain positive attitudes (23, 25, 27, 32, 34, 35, 37), they also exhibit caution and apprehension (29, 33–35, 37). The public expresses concerns about potential ethical issues arising from AI, thus placing high value on transparency and the right to informed participation (23, 27). They also emphasize the importance of human physician supervision, maintaining that humanistic care remains a core component of healthcare (33). The public is more supportive of AI assisting physician decision-making rather than replacing the technical roles of doctors (29, 35, 37). For example, studies by Rojahn et al. (29) and Witkowski et al. (35) demonstrate that the public prefers AI as a supportive tool for decision-making rather than a replacement for physicians. Research by Robertson et al. (37) reveals the complexity of patient attitudes, finding that acceptance of AI diagnosis is highly dependent on the specific clinical context; in cases involving serious illnesses or complex conditions, patient reliance and trust in physician judgment far exceed that in AI.

Owing to its spontaneity and real-time nature (38), the application of social media in researching the value of medical and health information is growing rapidly (39). In recent years, researchers have begun using social media to study public perceptions and attitudes toward specific healthcare technologies, such as non-invasive prenatal testing (40), COVID-19 vaccines (41), and medical consultations (42).

Currently, social media research focused on medical AI is still in its infancy (15–20), and conclusions remain divergent. Some studies indicate that public sentiment leans positive but is accompanied by concerns regarding privacy and unemployment risks (15, 18–20). For example, Cai et al. (16) analysis of mental health discussions on Reddit found that negative sentiments predominated and increased over time. A study centered on the Weibo platform suggested that while the public recognizes the potential for AI to replace physicians, trust in its technical reliability and associated companies remains low (17). Ocal (43) analysis of long-term Reddit discussions using the BERTopic model serves as a methodological template for this paper; the results showed that despite ethical concerns, the overall public attitude leaned toward curiosity and positivity.

Despite existing research yielding important conclusions and providing practical insights for AI developers and policymakers, limitations persist. On one hand, traditional survey methods face several constraints: first, sample sizes are typically limited to hundreds or thousands of participants and are restricted by fixed-option questionnaires, making it difficult to capture broader and more spontaneous public discourse (44); second, surveys and interviews reflect attitudes within a specific timeframe, making it challenging to track dynamic changes in opinion following major events or technological iterations over long periods (45); third, because the research agenda is usually set and controlled by researchers, such activities are termed “motivated dialogues” (46), and researcher guidance or questionnaire wording may induce response bias (46). On the other hand, existing social media research is insufficient: first, perspectives are fragmented, often focusing on specific technologies (such as imaging AI (15)) and lacking a comprehensive assessment of medical AI as a whole; second, methodological limitations exist, as most thematic analyses rely on manual qualitative coding (15, 17), which struggles to process massive volumes of text effectively, leading to inconsistencies and a lack of comprehensiveness in results. These limitations suggest the need for a scalable framework that can connect discussion themes, sentiment orientations, and temporal changes within the same analytical process.

This study aims to overcome these limitations through automated techniques, including machine learning, BERTopic-based topic modeling, large language model-based sentiment analysis, and temporal trend analysis. We selected Reddit as the research platform for several reasons: first, as a leading global social platform, its large active user base provides access to a wide range of public discussions (47); second, Reddit contains diverse health and technology sub-communities (Subreddits), facilitating precise data extraction and stratified analysis (48); third, unlike platforms primarily dependent on short-form content such as tweets, Reddit’s higher character limits allow users to engage in long-form discussions, providing high-quality material for refined sentiment analysis (49); fourth, Reddit has consistently served as a data source for various research designs and topics and has been used to study user engagement trends (50). Research indicates that Reddit is a reliable source for tracking public health concerns and information dissemination (49); fifth, compared to closed communities like Facebook, Reddit provides a more open and transparent research environment (51). Therefore, Reddit is used in this study not as a statistically representative sample of the global public, but as a suitable source for observing spontaneous, topic-rich, and temporally traceable online discourse on medical AI. Conceptually, this study interprets public attitudes through the lens of perceived usefulness, perceived risk, and trust, which are closely related to technology acceptance in healthcare contexts.

## 3 Materials and methods

### 3.1 Ethics statement

In the expanding field of Reddit research, ethical processing pathways primarily follow the “public domain approach” (52). This approach posits that publicly visible Reddit content constitutes public material, which researchers can generally utilize without undergoing traditional ethical review. Posts and comments on Reddit are highly public and accessible to anyone (53). Consequently, many studies employing such public social media data are not considered human subject research in the traditional sense (53, 54).

This study adheres to these principles while adopting more cautious ethical practices. We strictly followed the Reddit User Agreement in effect during the data collection period (55), which explicitly states that user-posted content is publicly displayed information and that users have pre-consented to third-party access and storage, provided terms are met. Accordingly, we only crawled publicly visible text, engaged in no direct interaction with users, and conducted no interventional experiments. Therefore, this study does not fall within the scope of research involving human subjects, complies with non-commercial academic use standards, and strictly upholds the core principles of the Association of Internet Researchers’ Ethical Guidelines (AoIR 3.0).

Furthermore, we recognize that mere compliance does not fully address the ethical complexities of online research. Researchers must seek a balance between academic freedom, platform regulations, and user expectations (56) while taking substantive measures to protect user privacy (57). To maximize user privacy protection, we implemented rigorous anonymization measures throughout the data processing workflow. All identifying information, such as usernames, was removed at the extraction stage, and data were analyzed in aggregate form. To prevent unnecessary attention or potential risk to users, any original comments cited in the manuscript were paraphrased semantically rather than quoted directly; these were subsequently edited and verified for non-traceability by the first author. To further safeguard the privacy of Reddit users, we sampled posts from a wide range of subreddits to mitigate the ethical risks associated with exposing specific sub-communities or their members (53).

Based on the aforementioned ethical considerations and specific practices, this study did not require independent ethical review or approval.

### 3.2 Search strategy

Following the methodology of Almanaa (15), this study employs a search strategy designed to capture a broad range of discussions related to AI in healthcare. The keywords utilized in the search include general terms such as AI, Artificial Intelligence, Healthcare, and Medicine; specific domains including medical diagnosis, mental health, radiology, automation, data analysis, telemedicine, and medical imaging; and cutting-edge technologies such as ChatGPT. Boolean operators OR and AND were applied to combine these keywords to ensure comprehensive coverage of relevant topics. The specific syntax for keyword queries followed a logical OR combination approach, such as (AI OR Artificial Intelligence) AND (Healthcare OR Medicine). In the implementation, the retrieval query combined AI-related terms, such as AI, Artificial Intelligence, Machine Learning, Deep Learning, Natural Language Processing, and Computer Vision, with healthcare-related terms, such as healthcare, medical, medicine, clinical, patient, diagnosis, treatment, hospital, radiology, pathology, genomics, digital health, healthtech, and medtech. Additionally, specific technical terms, including Telemedicine, Medical Imaging, or ChatGPT, were incorporated within certain sub-communities to enhance recall rates.

### 3.3 Data collection and cleaning

In this study, “Public” specifically refers to active users of Reddit’s English-speaking communities. This operational definition introduces platform and language bias, because non-English-speaking users and people who do not use Reddit are not represented in the dataset. Influenced by the platform’s ecosystem, different subreddits exhibit cultural stratification; for instance, technology-oriented subreddits (e.g., r/technology) emphasize innovation and efficiency, whereas medical ethics subreddits (e.g., r/Health) focus more on privacy and security. These cultural differences influence users’ expressions and emotional orientations regarding medical AI topics to a certain extent.

Regarding sampling strategy, we refer to existing research. Caplan and Purser (58) noted that community boundaries and units of analysis should be clearly defined before data crawling to ensure contextual consistency. Zapcic et al. (59) pointed out that studies on public attitudes must balance sample heterogeneity and community differences to avoid ideological biases from specific subreddits. Furthermore, Qin et al. indicated that when conducting social media research on open platforms like Reddit, researchers should consider data selection, sampling frameworks, and theory-driven perspectives (60). We acknowledge the value of these methods for in-depth qualitative research; however, since this study aims to present the overall public discourse on medical AI rather than a theory-driven analysis focused on specific issues, we employ large-scale, multi-community data crawling and unsupervised natural language processing methods to capture semantic patterns and sentiment distributions across communities. While this approach sacrifices some depth in reflexive analysis, it broadens the range of observed discussions, aligning better with the objectives of exploratory research.

In the data collection phase, we implemented a multi-community integrated sampling strategy to capture the diverse discourses shaped by different subreddit cultures, thereby broadening the range of observed discussions and improving the transparency of the analysis. Specifically, to collect as many discussions related to medical AI as possible, we referred to the study by Yan et al. (61) and selected posts specifically updated on AI in healthcare. 35 subreddits were identified for inclusion in this study (Table 1). More specifically, these subreddits (1) explicitly focus on medical AI (e.g., HealthAI, HealthcareAI, MedicalAI); (2) specifically address medical AI technologies (e.g., Telemedicine, DigitalHealth); or (3) focus on news and discussions regarding AI technology and science, which include various current AI-related conversations (e.g., worldnews, science, technology). We performed data collection by directly calling Reddit’s official Application Programming Interface (API) to ensure precise control over the retrieval process. Hypertext Transfer Protocol (HTTP) GET requests were sent to Reddit’s API endpoints, with each request containing the following key parameters: (1) Query parameter (q): set as a Boolean logic string, such as ‘(AI OR Artificial Intelligence) AND (Healthcare OR Medicine)’, to search within the specified 35 subreddits; (2) Sort option (sort): set to ‘new’; (3) Time range (t): set to ‘all’, combined with our script logic to filter posts published between March 1, 2020, and March 31, 2025; (4) Search limits and pagination: we set the results limit per page to the maximum value of 100. To collect all eligible posts, we programmatically handled the pagination logic by utilizing the ‘after’ identifier returned in each API result as the parameter for the subsequent request, looping until all relevant results were traversed.

**Table 1:** Information about subreddits.

Subreddit	Description of subreddit	Number of members	Score^1^
Worldnews	A central repository of major global news and events, encompassing coverage across politics, technology, health, and other key domains.	45.6 m	18.2 m
Science	A multidisciplinary platform for scientific communication, emphasizing peer review and reliable information.	33.9 m	13.6 m
Futurology	Explores trends and possibilities in technology, society, and the future of humanity.	21.6 m	8.6 m
Technology	Focuses on technology news and discussions, covering a wide range of topics from consumer electronics to cutting-edge innovations.	19.0 m	7.6 m
Singularity	Concentrated on themes including the technological singularity, transhumanism, and the future impact of AI on human society.	3.7 m	1.5 m
MachineLearning	Centers on topics such as technological singularity, transhumanism, and AI’s influence on humanity’s future.	3.0 m	1.2 m
Conspiracy	Discusses various conspiracy theories with diverse viewpoints, often involving skeptical perspectives on technology and health-related issues.	2.2 m	0.9 m
ArtificialInteligence	A discussion space on AI development, encompassing ethics, applications, and research trends.	1.4 m	0.6 m
Artificial	A public-oriented section for sharing AI news and trends, featuring broad content and inclusive of diverse perspectives.	1.1 m	0.4 m
DeepLearning	Focused on practical exchanges of deep learning techniques and frameworks, suitable for researchers and developers.	191 k	76.6 k
Automate	Focused on the application of automation technologies in industry, daily life, and digital systems.	142 k	56.9 k
PublicHealth	A specialized forum for discussions on public health policies, health trends, and global health events.	142 k	56.7 k
Bioinformatics	A gathering place for bioinformatics enthusiasts, covering topics such as genome analysis and data modeling.	133 k	53.1 k
Computervision	A specialized community focused on computer vision technologies, such as image recognition and object detection.	115 k	46.1 k
DarkFuturology	A philosophical community exploring potential negative impacts and apocalyptic scenarios arising from future technologies.	69 k	27.7 k
AGI	A forum for discussing the concepts, research, and potential impacts of general artificial intelligence (AGI).	63 k	25.2 k
HealthIT	Focused on healthcare information technology, including electronic medical record systems, interoperability, and security issues.	41 k	16.3 k
Genomics	Focuses on advancements in genomics research and its applications in disease studies and precision medicine.	17 k	7.0 k
Innovation	A hub for showcasing innovative products and ideas, emphasizing technology, business, and design.	16 k	6.3 k
MedTech	A community focused on medical technology news and product development, covering cutting-edge topics such as medical devices and AI applications.	6 k	2.4 k
Tomorrowsworld	A forum exploring visions of future technology and society, with an emphasis on science fiction, futurism, and macro trends.	4 k	1.7 k
HealthAI	A dedicated platform for in-depth discussions on the development, tools, and ethical issues of medical AI.	3 k	1.0 k
HealthTech	A communication hub for medical technology innovation, covering telemedicine, wearable devices, and intelligent diagnostics.	2 k	0.9 k
ComputationalBiology	A technical discussion forum at the intersection of computational modeling and biological data analysis.	2 k	0.8 k
Telemedicine	A community focused on telemedicine technologies, covering technical implementation, policy changes, and user experience.	2 k	0.8 k
DigitalHealth	Focused on the digital health ecosystem, including mobile health, AI diagnostics, and data interoperability.	2 k	0.7 k
DrugDiscovery	A professional community for drug development, discussing the role of AI in compound screening and target discovery.	1 k	0.4 k
HealthcareTechnology	A collection of the latest developments in the medical technology field, covering AI, hardware, and software tools.	1 k	0.4 k
HealthcareStartups	Topics related to healthcare entrepreneurship, including AI-driven health technology startups.	1 k	0.3 k
MedicalImaging	Focused on medical imaging technologies, including AI-based image analysis and diagnostic support systems.	1 k	0.3 k
EHealth	Topics related to electronic health services and policies, addressing both technological and social impacts.	0.4 k	0.2 k
PrecisionMedicine	A platform for exchanging precision medicine approaches and research findings, often involving AI and genomic data.	0.4 k	0.2 k
HealthData	Focusing on the management, analysis, and privacy protection of health data, including related technological and policy issues.	0.4 k	0.2 k
HealthDataScience	A professional discussion community on medical data science, covering statistical modeling and AI applications.	0.1 k	0.04 k
HealthcareAI	A specialized community with a deep focus on various AI applications in healthcare, ranging from algorithms to clinical deployment.	0.1 k	0.04 k

This process initially retrieved 4,041 independent posts. To address potential selection bias and ensure the representativeness of the final sample, we implemented a rigorous screening process rather than arbitrary sampling. The inclusion criteria were as follows: a post must (1) substantively discuss the application, impact, or public perception of AI in the medical field in the title or body; and (2) trigger meaningful public discussion, operationally defined as having at least five comments under the post to ensure that the retained posts contained sufficient interaction for comment-level sentiment and discussion analysis. Only posts meeting all these criteria were included in the final sample. The threshold of five comments was selected as a pragmatic minimum to exclude isolated or low-engagement posts while retaining discussions with enough user responses to reflect interaction beyond the original post.

Subsequently, we performed data preprocessing. Following the method of Yadav et al. (62), we cleaned the text by removing numbers, emojis, links, punctuation, non-ASCII characters, stop words, phrases, redundant spaces, and irrelevant words, utilizing the English stop word list provided by the Natural Language Toolkit (NLTK) (63). For word-frequency visualizations, common singular and plural variants were consolidated where appropriate to reduce redundant lexical forms. All sentences were converted to lowercase, tokenized, and de-accented. Tokenization refers to splitting text into smaller units for analysis, and de-accenting refers to standardizing characters by removing accent marks. To avoid translation errors, only English text was retained, and non-English tokens were removed based on a dictionary. We also identified and excluded duplicate entries and bot-generated content. Specifically, we employed a filtering method based on a cleaning keyword list, which was separate from the retrieval keyword list used during data collection. Our detection range included common subreddit-level moderation bots (e.g., accounts with the username “AutoModerator”) as well as a broader range of bots producing inauthentic content, such as accounts posting advertisements or spam. Our keyword list contained numerous commercial promotion terms and common patterns used to identify automated behavior, ensuring the authenticity of the user-generated content in the corpus. The cleaning process used two types of keyword filters: relevance filters, including AI-related terms such as AI, Artificial Intelligence, GPT, LLM, ChatGPT, GPT-4, AI-powered, and Chatbots, and healthcare-related terms such as Medical, Healthcare, Pathology, Diagnosis, Doctors, Patients, Cancer, and Radiology; and noise filters, including terms related to advertisements, scams, gambling, adult content, link bait, spam, and other irrelevant or automated content. The full retrieval and cleaning keyword lists are provided in the code repository and Supplementary material to improve transparency and reproducibility.

**Figure 1:**
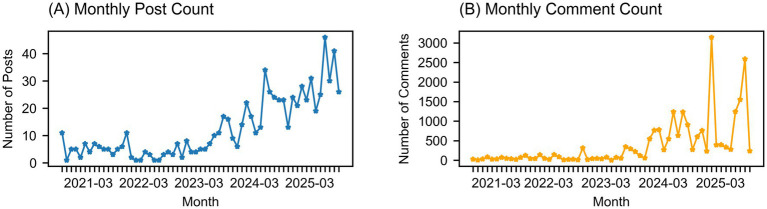
Monthly counts of Reddit posts and comments after screening. **(A)** Monthly Post Count. **(B)** Monthly Comment Count.

Ultimately, the initial 4,041 post records were refined into a final set of 1,020 posts, yielding 36,555 comments for analysis. To provide a clearer breakdown of data reduction, the screening process was summarized as follows. Of the 4,041 initially retrieved records, 976 were removed because the corresponding posts were unavailable, failed to download, or could not be parsed into complete JSON files, leaving 3,065 archived raw post files. A further 88 records were removed during preliminary cleaning because of duplication, inaccessibility, or structural incompleteness, leaving 2,977 posts. Relevance and quality screening then excluded 444 weakly relevant, non-English, or low-quality records, leaving 2,533 relevance-screened posts. Noise filtering further removed 1,419 promotional, bot-like, spam-like, or insufficiently analyzable records, leaving 1,114 eligible posts. Finally, 94 records were removed during final consistency checking, resulting in 1,020 posts in the final analytical corpus. Overall, 3,021 records were excluded, and the final post-level retention rate was 25.2%. Posts were excluded mainly because they were unavailable or structurally incomplete, duplicated previously collected records, lacked substantive relevance to medical AI, contained non-English or low-quality content, or showed signs of bot-generated, promotional, or spam-like activity. This reduction resulted from a systematic filtering process that applied an extensive keyword list to ensure thematic relevance and required each post to have at least five comments to filter for substantive discussion. Although this filtering process improved topical relevance and interaction quality, it may have excluded some low-engagement but relevant posts; this potential selection bias is acknowledged as a limitation. Figure 1 shows the monthly number of Reddit post submissions and comments after data screening for the period from March 1, 2020, to March 31, 2025. This figure is used to assess the temporal coverage of the final dataset and to show that the retained discussions were not evenly distributed over time, which provides context for the subsequent sentiment trend analysis. The increase in post and comment volume after 2023 also indicates that public discussion of medical AI became more active during the later part of the observation period.

### 3.4 Topic modeling

Topic modeling is widely used in text mining to summarize major themes in large text corpora (64). Most existing studies employ Latent Dirichlet Allocation (LDA) topic modeling to explore public attitudes on social media (41, 65). However, the limitations of this approach cannot be overlooked, including the neglect of semantic relationships between words (66), the complexity of the topic modeling process, a lack of independence and orthogonality between generated topics (67), and high demands on time and labor (19). Furthermore, when processing large-scale text data, LDA exhibits a deficient capacity to capture contextual information and limited robustness in handling sparse or noisy data (68).

To overcome these limitations, we selected the BERTopic (69) method. BERTopic was selected because it combines transformer-based semantic embeddings with density-based clustering and class-based Term Frequency-Inverse Document Frequency (c-TF-IDF) topic representation, making it suitable for short, informal, and semantically diverse social media texts. Research indicates that BERTopic often outperforms traditional probabilistic models, such as LDA or Non-negative Matrix Factorization (NMF), in terms of topic coherence and interpretability (63). Theoretically, it is better suited for capturing subtle semantic nuances in the short, informal, and highly context-dependent texts common on social media (66). To reduce the influence of stochastic variation, we examined whether the major topic structure and high-weight keywords remained consistent across repeated model runs and parameter adjustments during model refinement. These results provide quantitative evidence that BERTopic produced meaningful and coherent topics for our specific dataset compared to traditional models. This framework is based on the Bidirectional Encoder Representations from Transformers (BERT) model (70) and incorporates Sentence-BERT (SBERT) embeddings (71) along with Hierarchical Density-Based Spatial Clustering of Applications with Noise (HDBSCAN) (72), which is used to cluster the reduced embeddings and identify outliers. It extracts topic words using c-TF-IDF (73), which not only addresses the limitations of LDA but also provides advanced functionalities such as search, hierarchical, and dynamic topic modeling (19). The topic modeling pipeline used the all-MiniLM-L6-v2 sentence-transformer model for document embeddings and a CountVectorizer with English stop-word removal and unigram/bigram features for topic representation. The specific workflow is as follows:

1 Document embedding: this study utilizes the all-MiniLM-L6-v2 pre-trained model to map text into a 384-dimensional vector space. This vector space is a mathematical representation where text is converted into numerical coordinates; each of the 384 “dimensions” represents an abstract feature of the text’s meaning, allowing the model to mathematically calculate and compare semantic similarities between different documents. Building on this, c-TF-IDF is further integrated to generate topic word distributions for each cluster (69).
2 Dimensionality reduction: uniform manifold approximation and projection (UMAP) is employed to reduce dimensions while preserving both the local and global structures of the data, thereby enhancing processing efficiency. Unless otherwise specified, the BERTopic default UMAP configuration was used to maintain consistency with the standard implementation.
3 Clustering: the default HDBSCAN configuration was retained because it allows the model to identify clusters of varying density and assign noisy documents to outliers without predefining the number of topics.
4 Bag-of-words representation: documents within each cluster are merged, and word frequencies are calculated to construct a bag-of-words representation.
5 Topic representation: c-TF-IDF is utilized to extract distinguishing keywords from the categorized text, generating more precise topic features.

This five-step process links semantic clustering with keyword-based topic representation, making the resulting topics easier to interpret. In plain terms, this workflow first converts text into semantic vectors, then groups semantically similar texts, and finally extracts representative words to describe each group.

To validate the quality of our final topic structure, we evaluated two key quantitative metrics: topic cohesion and topic separability. Topic cohesion reflects whether the representative words within the same topic are semantically related, whereas topic separability reflects whether different topics are sufficiently distinct from one another. We used the Normalized Pointwise Mutual Information (NPMI) score to measure topic cohesion, because NPMI is commonly used to evaluate whether the top words within a topic tend to co-occur and form a coherent semantic unit. Our model achieved an NPMI score of 0.65, indicating relatively high internal coherence for the extracted topics. To assess topic separability and address potential topic drift, we calculated the average cosine similarity between all topic pairs, with lower average similarity indicating clearer semantic separation among topics. An average similarity below 0.3 indicates that the individual topics are clearly distinguishable within the semantic space. Together, these quantitative metrics confirm that the 14-topic structure we ultimately identified possesses strong cohesion, separability, and interpretability.

### 3.5 Sentiment analysis

Sentiment analysis has garnered extensive attention in the research community in recent years, particularly with its rapidly increasing application within the healthcare sector (74). Analyzing data generated by patients on social media facilitates the provision of enhanced healthcare services (74, 75). Sentiment analysis is a supervised classification task that categorizes sentences into positive, negative, or neutral classes based on the emotional tone conveyed by the text.

Given the complexity and nuances of healthcare discussions on social media, traditional sentiment analysis methods based on lexicons or small-scale models face limitations when processing context, irony, and negations. To achieve more accurate and context-aware sentiment classification, we adopted an advanced Large Language Model (LLM) approach (76). Specifically, we used DeepSeek Chat (deepseek-chat) through an OpenAI-compatible API for zero-shot sentiment classification, with JSON output enabled and default generation parameters retained. Zero-shot sentiment classification refers to assigning text to predefined labels without task-specific model training. For each comment, we designed a structured prompt to guide the model output. The prompt required the model to determine whether the emotional tone was positive, negative, or neutral based on both the individual comment and its parent context. The system prompt instructed the model to act as an AI healthcare sentiment analysis expert and to return the sentiment label (Positive, Neutral, Negative, or Meaningless), a topic label, and a relevance score in JSON format. The user prompt included the item ID, text type, post title, post body, parent comment, and comment body, enabling the model to evaluate each entry with its conversational context. A representative prompt template is provided in the Supplementary material to support reproducibility. This approach effectively mitigates biases that arise when a model analyzes the sentiment orientation of a comment in isolation. Compared to traditional classifiers that require task-specific fine-tuning, such as RoBERTa, the zero-shot classification capability of the LLM better handles sarcasm, ambiguity, and complex medical discourse. This is because the LLM leverages reasoning based on patterns learned from massive datasets rather than merely matching keywords or simple grammatical structures. This method supported more context-aware sentiment classification and provided a practical basis for the sentiment analysis in this study.

### 3.6 Sentiment trend analysis

Sentiment trend analysis is useful for examining how public attitudes toward specific issues change over time (74). In this study, we utilized a multifaceted approach combining BERTopic topic modeling with sentiment analysis to examine emotional trends in public sentiment regarding AI in healthcare on Reddit, covering the period from March 1, 2020 to March 31, 2025. Because this period includes the public release of ChatGPT in November 2022 and the release of GPT-4 in March 2023, the temporal analysis was interpreted with attention to the broader diffusion period of generative AI rather than a single isolated event.

First, we constructed a comprehensive data framework containing key informational dimensions, including timestamps, sentiment polarity labels, topic categories, and monthly sentiment scores. The timestamps were normalized into a standard datetime format. For each entry, sentiment polarity was coded as +1 for positive, 0 for neutral, and −1 for negative; the monthly sentiment score was calculated as the average coded sentiment within each month. By applying finely tuned thresholds to sentiment scores, we mapped sentiment values into three semantic categories: “positive,” “negative,” and “neutral,” thereby enabling a multilayered quantification of public attitudes. Second, during the data aggregation phase, we grouped entries by month and sentiment type to calculate the count and proportion of each sentiment category within each time unit. To visually present the dynamic evolution of sentiment, we used the matplotlib visualization tool to generate stacked area charts that intuitively depict the temporal trajectory of public attitudes toward AI in healthcare. This visualization strategy enhanced the readability of the data and provided readers with a comprehensive and nuanced view of sentiment trends. Thus, the long-term trend was assessed using two descriptive indicators: monthly sentiment proportions and the monthly average sentiment score, which together illustrate how Reddit users’ expressed emotions regarding AI in healthcare changed over time.

### 3.7 Validation methods

We conducted manual validation of the experimental results, focusing on two primary dimensions: topic modeling and sentiment analysis:

1 Topic modeling: to evaluate the accuracy of the generated topics, two independent researchers assessed 20 randomly selected comments for each topic, independently judging whether the comment content was consistent with the corresponding topic labels and keywords. The results indicated that over 90% of the comments were consistently identified as highly relevant to their assigned topics, confirming that our topic model effectively identifies meaningful and coherent discussion themes.
2 Sentiment analysis: to verify the reliability of our automated sentiment classification, two independent researchers performed manual labeling of a randomly selected sub-sample of 500 comments (approximately 2.7% of the dataset) into positive, negative, and neutral categories to construct a gold standard dataset. Inter-rater reliability was high (Cohen’s Kappa = 0.82). Subsequently, we compared the model’s classification results against this gold standard, achieving an accuracy of 85% and a macro F1-score of 0.84, which indicates that our sentiment analysis method possesses sufficiently high reliability. These validation results provide quantitative evidence for the reliability of the LLM-based sentiment classification used in the main analysis.

## 4 Results

The Results section presents descriptive findings from the topic modeling, sentiment classification, and temporal analysis. Broader interpretation and implications are addressed in the Discussion section.

### 4.1 Topic modeling

**Figure 2:**
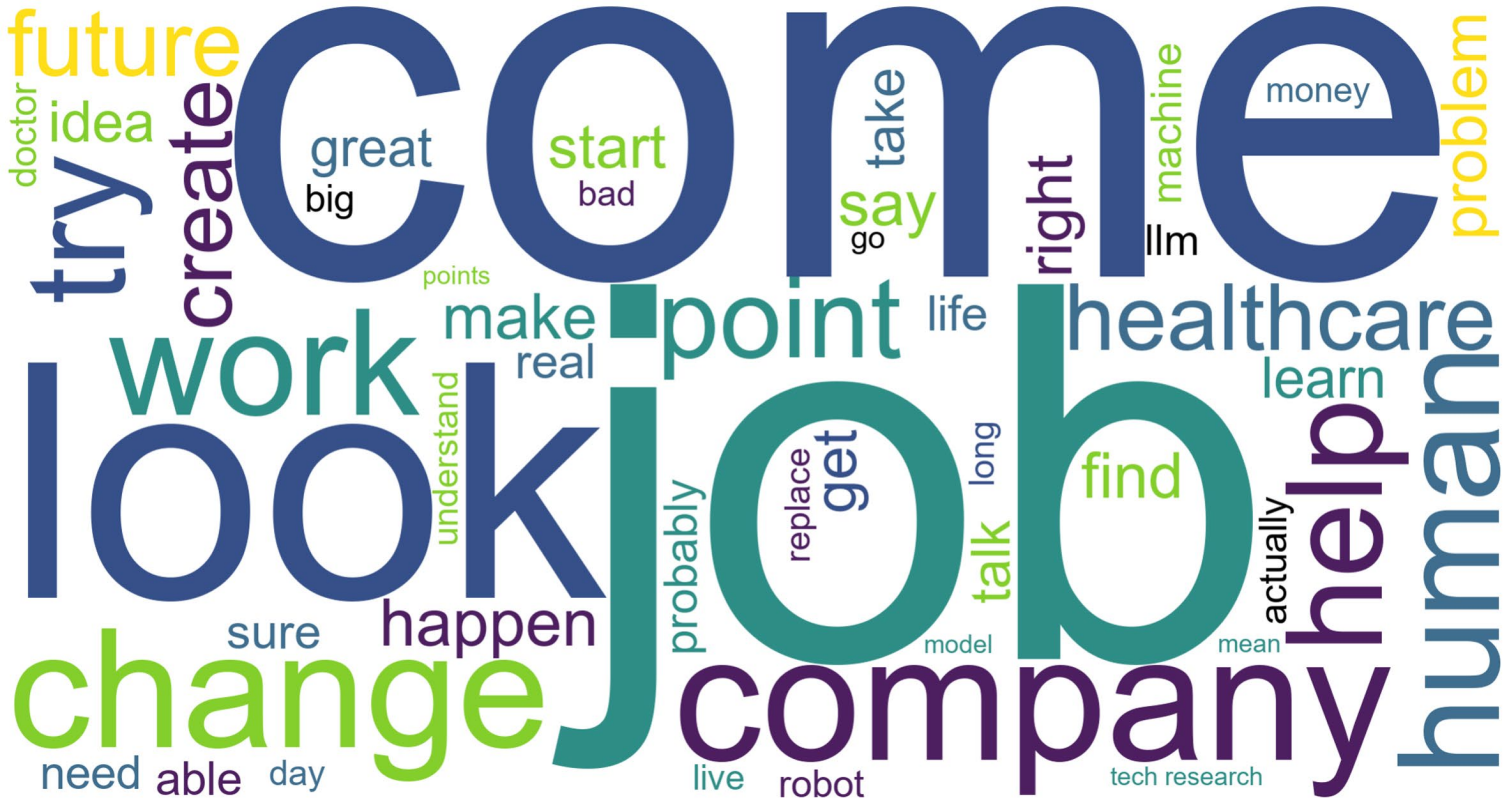
Word cloud of “AI in healthcare” on Reddit.

Reddit users discuss the applications of AI in healthcare by sharing slightly optimistic perspectives based on sentiment analysis results. Before diving into the in-depth BERTopic topic modeling results, we first present a preliminary visualization of the dataset using a word cloud. Specifically, the words in the word cloud are ranked by frequency, with a minimum font size of 4; the rendering process terminates automatically when space is insufficient for smaller fonts. Colors are assigned randomly to distinguish visual hierarchy, and font size is proportional to frequency. Figure 2 displays the word cloud generated from the comment content. High-frequency terms such as “work,” “help,” “create,” and “change” reflect public recognition and expectations for AI applications in healthcare. Simultaneously, the prominent distribution of words like “problem,” “doctor,” and “human” suggests that while the public anticipates medical AI, they maintain skepticism and distrust regarding risks, such as the potential replacement of human physicians.

**Figure 3:**
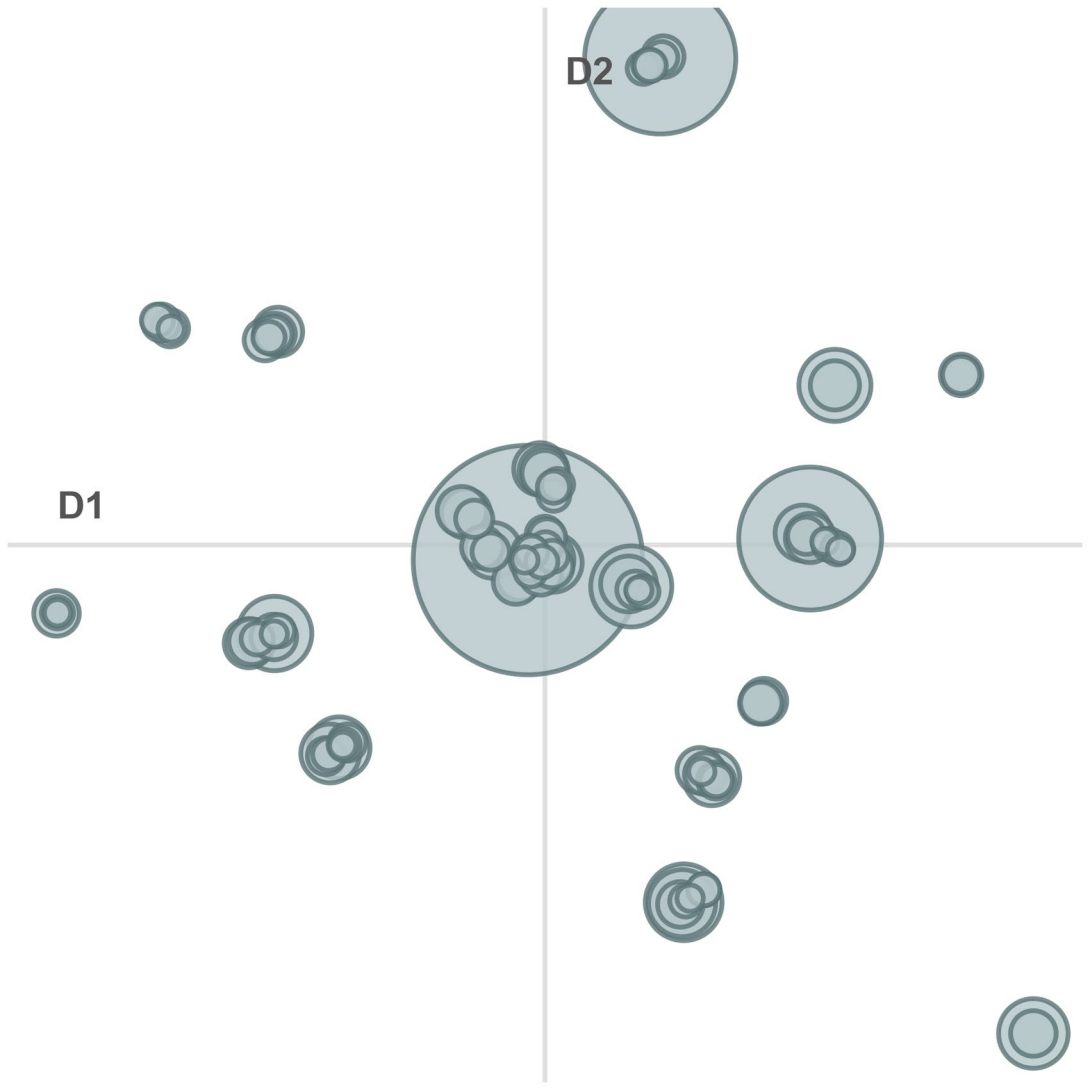
Algorithmically generated topic distribution. This figure illustrates the distribution of the algorithm-generated topics within a two-dimensional scaled space. Each circle in the plot represents a specific sub-topic, with its size generally corresponding to the number of documents associated with that topic. The spatial positioning of the circles reflects the relative relationships between topics: circles located in close proximity indicate topics with similar thematic content, whereas those separated by greater distances represent topics with substantial semantic differences.

This study employed the BERTopic algorithm, utilizing SBERT, UMAP, HDBSCAN, and c-TF-IDF modules for modeling. Initial modeling was performed with default settings. To further minimize noise, we then applied the reduce_outliers algorithm. reduce_outliers is a built-in BERTopic feature that reassigns documents initially identified as outliers to their semantically most similar topic clusters, thereby reducing data loss and improving topic coherence. Without pre-setting the number of clusters, the model automatically generated 97 topics. As shown in Figure 3, the overall topic distribution is characterized by small-scale aggregation and large-scale dispersion, indicating that further merging of small clusters is achievable.

**Figure 4:**
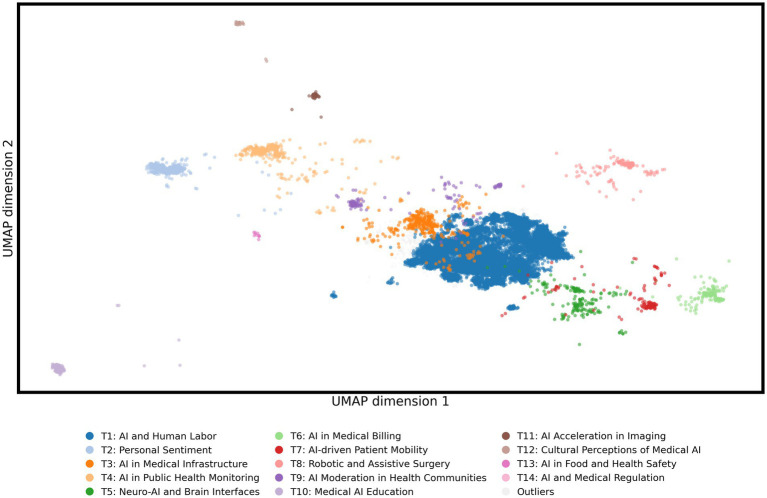
2D distribution of subtopic documents and their themes. This figure presents the distribution of documents associated with various subtopics within a two-dimensional space, offering a visual depiction that highlights clustering patterns and spatial relationships. This visualization aids in analyzing potential links or distinctions between different subtopics. Distinct subtopics are represented by colored regions, accompanied by a numbered list below that details each one. The positioning of these colored areas reflects thematic correlations; closely related topics cluster within neighboring regions, whereas clusters positioned farther apart indicate substantial thematic divergence.

**Figure 5:**
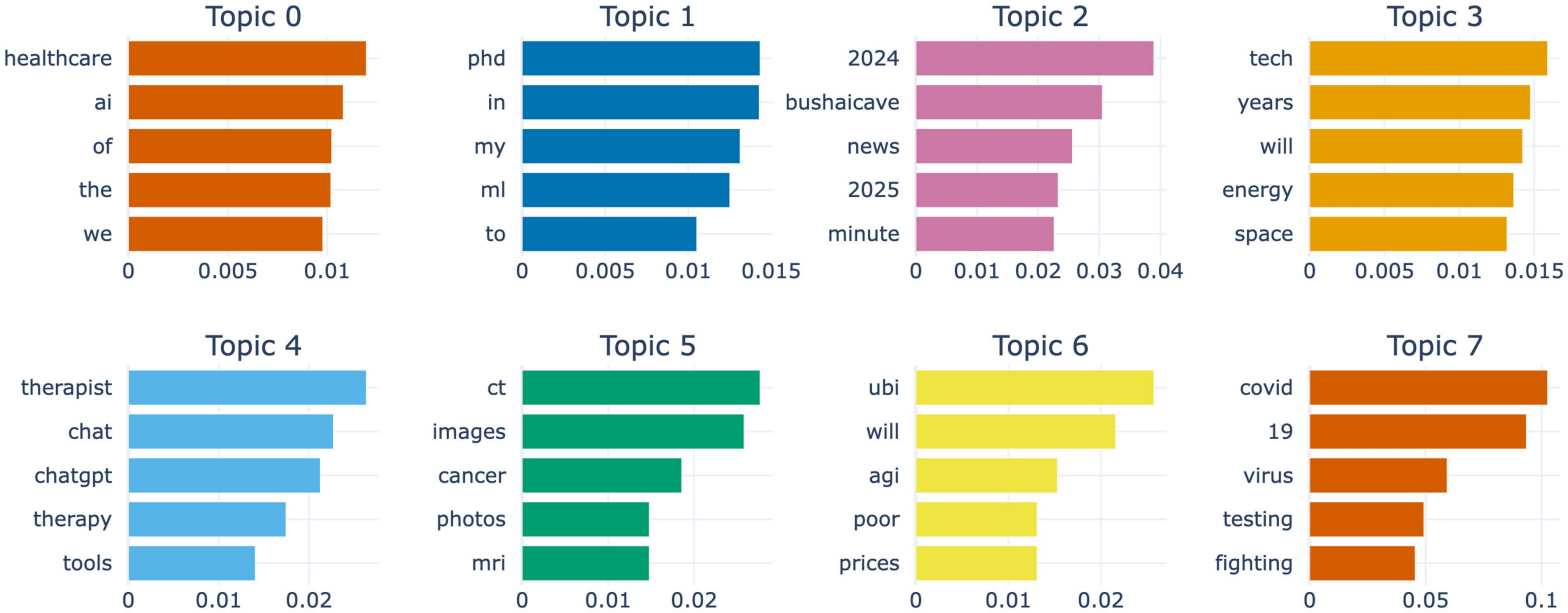
Contribution of keywords to subtopics. This figure provides a detailed breakdown of each theme by clearly emphasizing the core keywords in every subtopic. Each subplot represents a particular theme, with the horizontal axis showing the weight or importance of keywords and the vertical axis listing the top-ranking keywords by weight. The length of each bar reflects the significance of the keyword, where longer bars denote higher representativeness and a stronger impact on the theme’s content.

In the process of refining the number of topics, we manually reviewed, optimized, and aggregated the clusters. By iteratively adjusting BERTopic parameters, we determined that 38 topics served as an appropriate level for secondary topics. Figure 4 illustrates the spatial distribution of documents and their respective topics; a smaller spatial distance between documents or topics indicates higher thematic similarity. Figure 5 displays the top 8 topics and the keywords with the highest c-TF-IDF scores for each.

**Figure 6:**
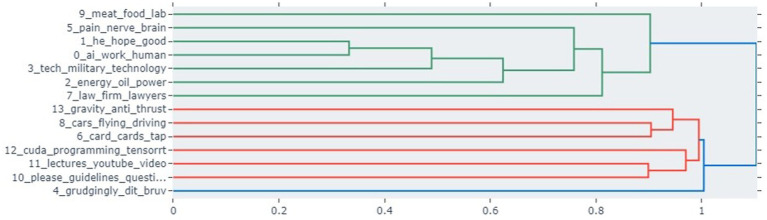
Hierarchical diagram of subtopics. This figure illustrates the relative relationships and hierarchical structure among the extracted subtopics. On the left side, theme numbers and brief descriptions are listed. Colored lines indicate different thematic categories and their clustering branches, helping to identify broader groupings within the topics. The horizontal axis measures the degree of content similarity between themes: lower values indicate closer content connections between linked themes, whereas higher values reflect greater differences in content.

**Figure 7:**
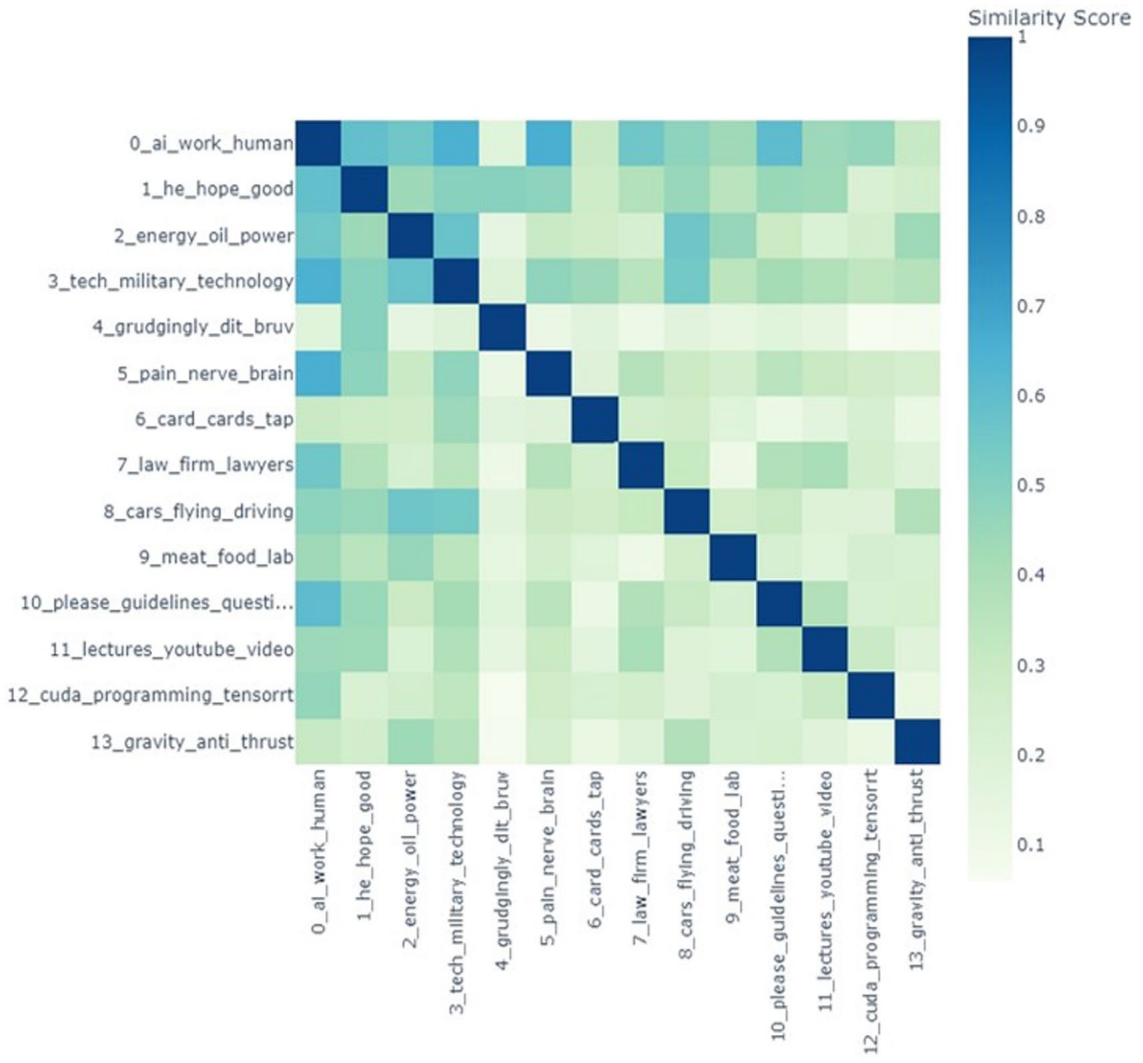
Similarity matrix of subtopics. This figure depicts the similarity relationships among the subtopics. The heatmap uses color intensity to represent the degree of similarity between themes: darker colors indicate higher content similarity, whereas lighter colors suggest greater content differences.

The secondary topic hierarchy (Figure 6) visualizes the hierarchical clustering relationships among the 14 topics in a dendrogram, where identical colors indicate high semantic similarity. The corresponding topic similarity matrix (Figure 7) presents pairwise similarities quantitatively. Together, Figures 6, 7 provide visual and data support for topic categorization from both structural and numerical perspectives. Furthermore, we integrated these visualizations to evaluate inter-topic similarity and reveal latent semantic associations. To interpret these similarity values, we developed a scoring system (Table 2) to quantify the degree and significance of similarity, facilitating the identification of mergeable topics.

**Table 2:** Meaning and explanation of topic similarity scores.

Similarity score range	Explanation
1.0	Identical: The content of the two topics is almost completely the same.
0.7–0.9	Highly Similar: The topics may belong to the same semantic category or closely related core concepts.
0.4–0.7	Moderately Similar: The topics are semantically related but differ in focus or application direction.
0.2–0.4	Low Similarity: The topics may occasionally share a small number of words but differ greatly in meaning and emphasis.
0–0.2	Almost Dissimilar: The topics belong to completely different semantic domains with virtually no substantive connection.

Results indicated that “AI and Human Labor” and “AI in Public Health Monitoring” shared a similarity of 0.65, showing high consistency. This indicates a significant overlap in discussions regarding the role of AI in human labor, specifically its replacement and synergistic effects within public health monitoring, leading to their categorization under the same theme. “AI in Public Health Monitoring” and “Neuro-AI and Brain Interfaces” showed a similarity of 0.49. While not meeting the threshold for full merging, these topics share semantic links regarding health sensing and data monitoring, specifically in physiological signal acquisition and health assessment. Additionally, a similarity of 0.46 among “Medical AI Education,” “Personal Sentiment,” and “AI in Medical Infrastructure” indicates a shared focus on the core issue of educational outreach and public acceptance during AI implementation in medical systems. Finally, “AI Moderation in Health Communities” and “Medical AI Education” had a similarity of 0.42, reflecting an overlap in the semantic space regarding AI content regulation and user education mechanisms.

**Figure 8:**
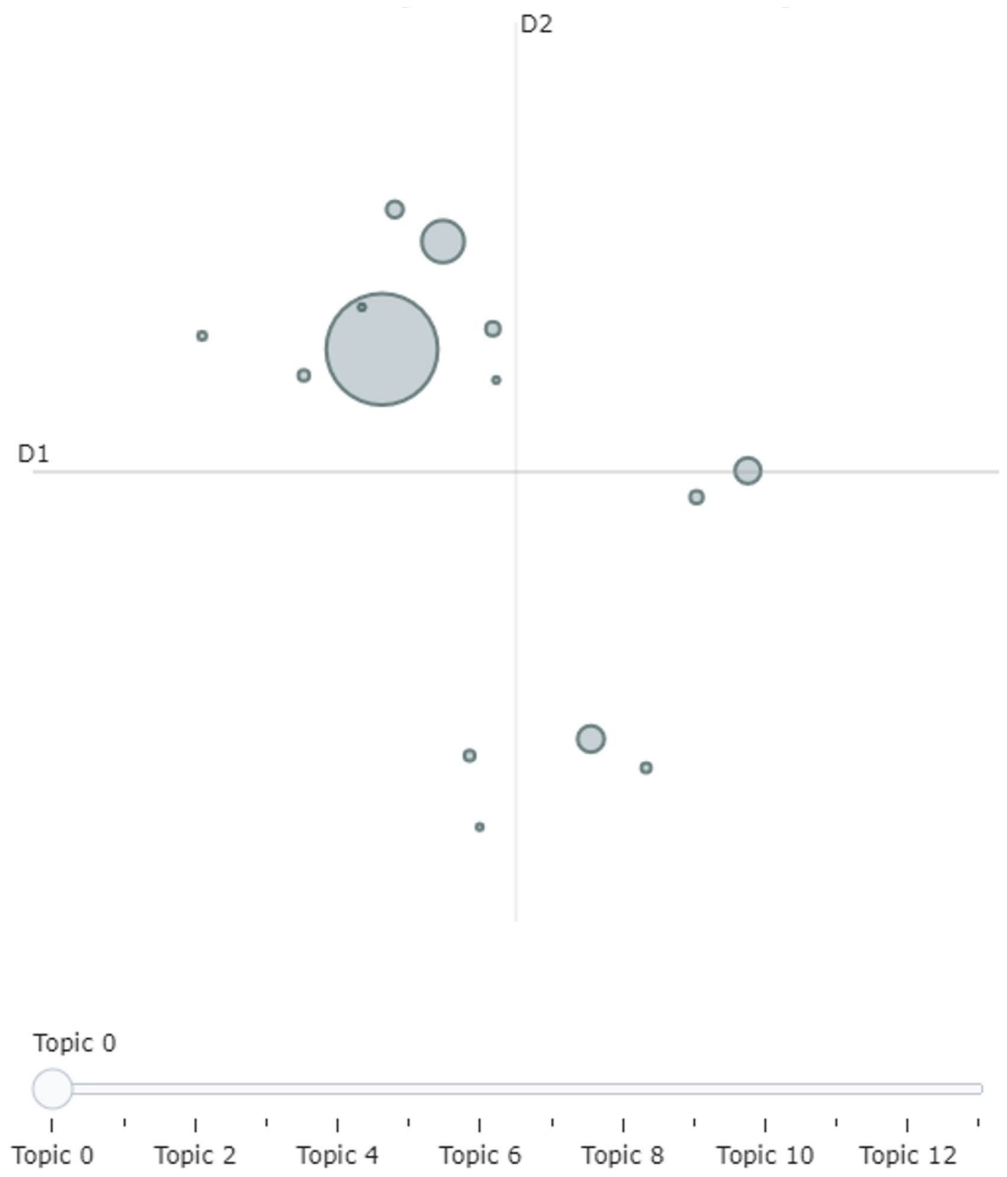
Distribution of subtopics. This figure illustrates the spatial distribution of subtopics within a two-dimensional scaled space.

After reviewing each topic structure, we performed manual topic merging and model updates using BERTopic, ultimately determining 14 secondary topics based on three criteria: semantic similarity between topics, interpretability of the top keywords, and consistency between topic labels and representative posts. The secondary topic distribution map (Figure 8) shows that the distribution of each topic is relatively dispersed with minimal local overlap, indicating an ideal clustering effect.

**Figure 9:**
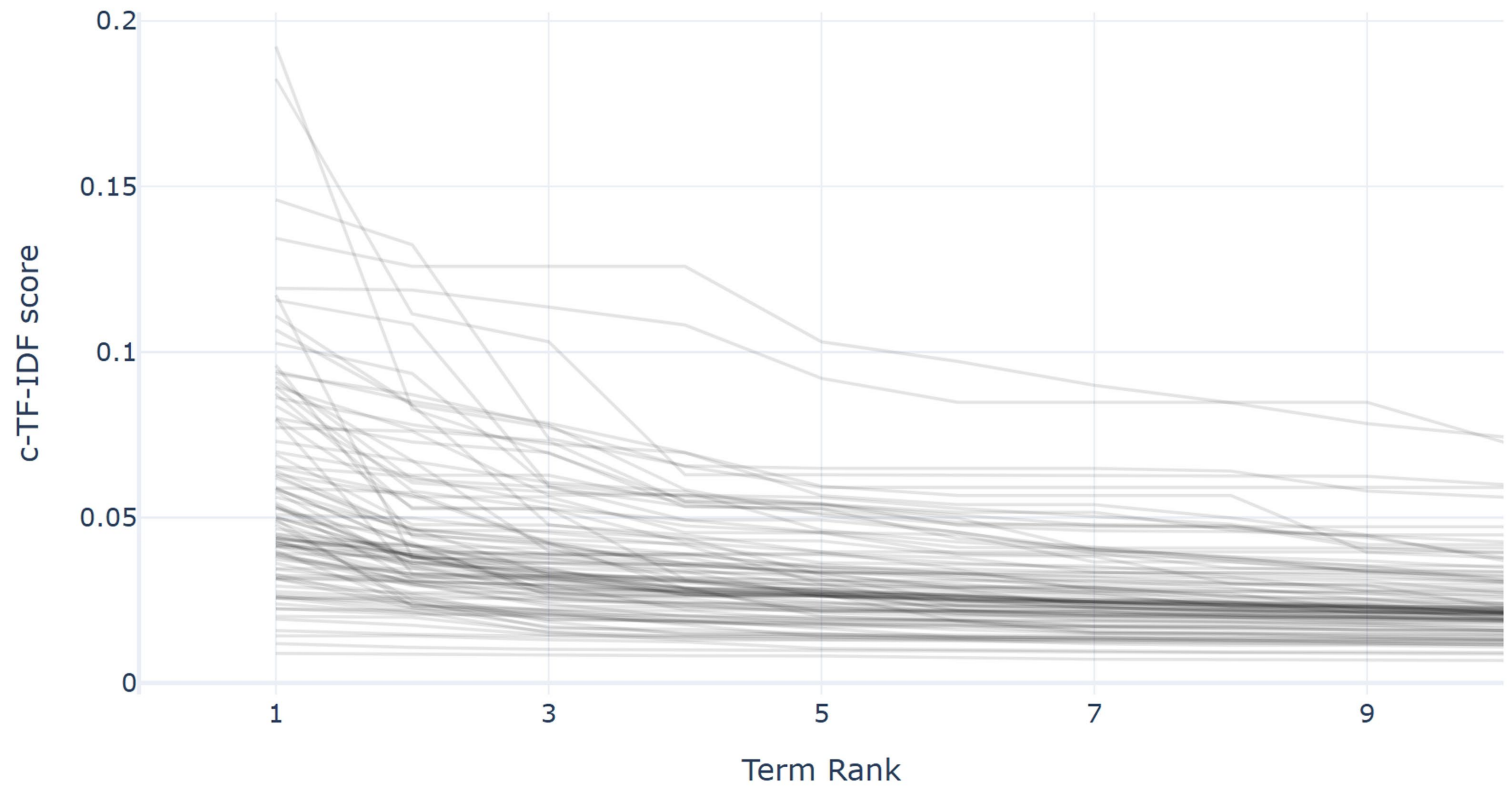
Line chart of keyword contributions for subtopics. This figure shows the trends in keyword contribution scores across rankings for each subtopic. Each curve corresponds to a specific subtopic, illustrating how the contribution of keywords, which is measured by c-TF-IDF scores, varies according to their rank. The horizontal axis, labeled “Term Rank,” orders keywords within the subtopic from highest to lowest contribution, while the vertical axis, “C-TF-IDF score,” indicates the contribution level of each keyword to the subtopic.

In naming each topic, we referenced the keyword information provided by the model. Each topic consists of high-weight terms where higher c-TF-IDF values signify a greater contribution to the topic’s semantic features. Keywords are ranked by their c-TF-IDF scores. Figure 9 illustrates the c-TF-IDF distribution of representative words for each topic, with the horizontal axis representing word rank and the vertical axis representing the c-TF-IDF score. The top three keywords generally summarize the core semantics of the topic.

To ensure objectivity and accuracy in naming, we adopted an iterative inductive approach. Two researchers independently examined the top 20 keywords and representative comments for each topic to propose descriptive labels. Discrepancies were resolved through discussion to reach a consensus on names that best encapsulate the core content. This procedure was also used to reduce subjectivity in topic merging, as topics were not merged solely on the basis of manual judgment but were checked against similarity scores, keyword distributions, and representative documents. This framework ensures that topic names are grounded in the data rather than subjective speculation.

To further validate semantic consistency and interpretability, we compiled representative posts for each topic (Table 3), with Reddit user comments semantically paraphrased to maintain anonymity. This table demonstrates typical user expressions, reflecting the semantic aggregation characteristics of each topic. These excerpts verify the content consistency and explanatory validity of the model’s topics.

**Table 3:** Representative paraphrased posts corresponding to BERTopic themes.

Topic ID	Topic name	Representative post excerpt
T1	AI and Human Labor	‘AI acts like a super-assistant for radiologists, screening out numerous normal images so we can focus on complex cases. However, it’s only a matter of time before some repetitive jobs are replaced.’
T2	Personal Sentiment	‘I used an AI diagnostic app that misdiagnosed my rash as skin cancer. AI has great potential, but it’s not mature enough to be fully trusted yet.’
T3	AI in Medical Infrastructure	‘The hospital’s use of AI for optimizing schedules was effective, but a single system update failure led to chaos, revealing the risks of over-reliance.’
T4	AI in Public Health Monitoring	‘Using AI to predict epidemic trends from social media data is a good approach, but the monitoring data is susceptible to noise, which could lead to false alarms and wasted resources.’
T5	Neuro-AI and Brain Interfaces	‘Brain-computer interfaces offer immense hope for paralyzed patients, but the potential misuse of reading or even writing thoughts poses a fundamental challenge to human free will.’
T6	AI in Medical Billing	‘My insurance claim was rejected because an AI system used the wrong medical code. Now I have to spend hours correcting a robot’s error.’
T7	AI-driven Patient Mobility	‘While autonomous wheelchairs are convenient, can their responsiveness in an emergency match that of a human caregiver?’
T8	Robotic and Assistive Surgery	‘My wife’s robotic surgery went well, but I’m still anxious about a machine operating inside her.’
T9	AI Moderation in Health Communities	‘In my chronic disease forum, an AI moderator deleted a post where we were asking for help and sharing experiences due to overly aggressive keyword filtering.’
T10	Medical AI Education	‘Medical students find using ChatGPT for their studies highly efficient, but educators worry that over-reliance might diminish their critical thinking skills.’
T11	AI Acceleration in Imaging	‘AI can dramatically reduce image reconstruction times, but the results still require careful review by human experts to rule out potential artifacts.’
T12	Cultural Perceptions of Medical AI	‘Here in the U.S., I feel the discussion around medical AI is more open. My friends in Europe, however, seem more wary of large corporations using AI to handle their health data.’
T13	AI in Food and Health Safety	‘Using AI to identify counterfeit drugs is an incredibly valuable application. However, if the algorithm is trained on biased data, it could be ineffective or even make false identifications in other regions.’
T14	AI and Medical Regulation	‘When an AI model continuously updates itself through online learning, is it still the same medical device that was initially approved? Regulations are clearly lagging behind.’

Based on this analysis, we named the primary and secondary topics and matched them with their corresponding keywords (Table 4). Public discussion on medical AI can be categorized into 6 primary themes and 14 secondary topics. The leading primary theme is “AI and Human-Centered Healthcare” (71.54%), focusing on how AI impacts human roles and work patterns. Discussions within this theme include AI and human labor (55.11%), personal sentiment toward AI (7.89%), basic applications of AI in medicine (3.12%), public health monitoring (2.96%), and specific medical AI technologies (2.46%). The second theme, “AI in Auxiliary Medical Services” (6.15%), focuses on enhancing efficiency and quality, covering AI medical payments (2.30%), patient-driven AI (2.07%), and robot-assisted surgery (1.78%). The third theme, “Platforms and Tools for Medical AI” (5.49%), addresses AI’s prevalence in medical systems and daily health management, including AI in health communities (1.91%), medical education (1.79%), and medical imaging (1.79%). The fourth is “Cultural Perceptions of Medical AI” (2.81%), focusing on the cognitive acceptance of the technology. The fifth is “AI in Food and Health Safety” (2.00%), addressing AI in pharmaceutical and food safety. The sixth is “AI and Medical Regulation” (2.12%), focusing on legal risks and regulatory issues in the medical application of AI.

**Table 4:** Topic information extracted by the BERTopic algorithm (*N* = 36,555).

Primary topic	Secondary topic	Topic keywords	Percentage
AI and human-centered healthcare	AI and human labor	[‘AI’, ‘work’, ‘human’, ‘time’, ‘up’, ‘data’, ‘which’, ‘most’, ‘only’, ‘medical’]	55.11%
	Personal sentiment	[‘He’, ‘hope’, ‘good’, ‘been’, ‘up’, ‘feel’, ‘very’, ‘here’]	7.89%
	AI in medical infrastructure	[‘Energy’, ‘oil’, ‘power’, ‘nuclear’, ‘solar’, ‘fusion’, ‘water’, ‘fuel’, ‘car’, ‘fossil’]	3.12%
	AI in public health monitoring	[‘Tech’, ‘military’, ‘technology’, ‘internet’, ‘were’, ‘been’, ‘us’, ‘public’, ‘time’]	2.96%
	Neuro-AI and brain interfaces	[‘Pain’, ‘nerve’, ‘brain’, ‘neuralink’, ‘chronic’, ‘hallucinations’, ‘vision’, ‘eye’, ‘hope’, ‘meds’]	2.46%
AI in auxiliary medical services	AI in medical billing	[‘Card’, ‘cards’, ‘tap’, ‘bank’, ‘transfers’, ‘debit’, ‘credit’, ‘pay’, ‘payment’, ‘chip’]	2.30%
	AI-driven patient mobility	[‘Cars’, ‘flying’, ‘driving’, ‘car’, ‘driver’, ‘waymo’, ‘helicopters’, ‘missouri’, ‘drivers’]	2.07%
	Robotic and assistive surgery	[‘Gravity’, ‘anti’, ‘thrust’, ‘propulsion’, ‘asymmetric’, ‘capacitors’, ‘keep’, ‘magnetic’, ‘antigravity’, ‘manipulation’]	1.78%
Platforms and tools for medical AI	AI moderation in health communities	[‘Please’, ‘guidelines’, ‘questions’, ‘post’, ‘artificialintelligence’, ‘gateway’, ‘mods’, ‘characters’, ‘bot’, ‘discussion’]	1.91%
	Medical AI education	[‘Lectures’, ‘youtube’, ‘video’, ‘lecture’, ‘videos’, ‘summaries’, ‘link’, ‘summary’, ‘able’, ‘dense’]	1.79%
	AI acceleration in imaging	[‘Cuda’, ‘programming’, ‘tensorrt’, ‘optimization’, ‘kernels’, ‘hardware’, ‘ml’, ‘custom’, ‘nets’, ‘working’]	1.79%
Cultural perceptions of medical AI		[‘Grudgingly’, ‘DIT’, ‘bruv’, ‘bizzare’, ‘thanks’, ‘merry’, ‘ambiguous’, ‘christmas’, ‘yah’, ‘what’]	2.81%
AI in food and health safety		[‘Meat’, ‘food’, ‘lab’, ‘eat’, ‘grown’, ‘protein’, ‘farms’, ‘foods’, ‘shrimp’, ‘agriculture’]	2.00%
AI and medical regulation		[‘Law’, ‘firm’, ‘lawyer’, ‘case’, ‘AI’, ‘cases’, ‘attorneys’, ‘briefs’, ‘legal’]	2.12%

### 4.2 Sentiment analysis

We categorized the emotional tone of the entries into three classes: positive, neutral, and negative. Across all comments and posts, analysis revealed that approximately 41.4% of entries conveyed positive sentiment. In contrast, approximately 35.1% expressed negative sentiment, while neutral sentiment accounted for the smallest proportion at approximately 23.8%.

**Figure 10:**
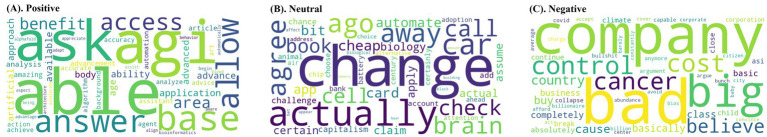
Word clouds of high-frequency words across sentiment categories: **(A)** Positive, **(B)** Neutral, **(C)** Negative.

TF-IDF values serve as a descriptive metric for measuring term distinctiveness in text mining. We conducted a visualization analysis of high-frequency words across different sentiment categories (Figure 10). Building on this, we selectively displayed representative high-frequency words and their corresponding TF-IDF scores within each sentiment category (Table 5) to describe the linguistic features associated with different sentiment categories. The results indicate that in positive discussions, terms related to the technical advantages of AI in healthcare were prominent. As shown in Figure 10A, among high-frequency words expressing positive sentiment, “able,” “benefit,” “access,” and “ability” were mentioned frequently. In Table 5, terms such as “help,” “find,” “able,” “doctor,” and “create” exhibited high TF-IDF scores, indicating strong discriminative power within positive contexts. These findings suggest that positive discussions often mention AI’s contributions to the medical field, such as assisting physicians, supporting auxiliary care, and improving diagnostics. Users perceive that medical AI can not only support the work of human doctors but also drive progress in medical technology. Furthermore, another primary driver of positive attitudes is optimism regarding the future development of medical AI, as evidenced by terms like “future” and “hope” in Table 5, which reflect public confidence and expectations for the industry’s growth.

**Table 5:** High-frequency words and their TF-IDF values across different sentiment.

Positive		Neutral		Negative	
Example	TF-IDF	Example	TF-IDF	Example	TF-IDF
Help	0.1497	Point	0.1518	Job	0.1935
Look	0.1321	Say	0.1468	Problem	0.1459
Find	0.1143	Mean	0.139	Bad	0.1362
Healthcare	0.1129	Come	0.1385	Happen	0.133
Doctor	0.1032	Right	0.1213	Get	0.1324
Research	0.1006	Work	0.1208	Company	0.1271
Create	0.0979	Change	0.118	Make	0.1241
Future	0.0959	Start	0.1025	Long	0.1165
Model	0.0906	Tech	0.1025	Human	0.115
Learn	0.0881	Actually	0.0991	Money	0.1085
Able	0.087	Different	0.0853	Life	0.0997
Great	0.0869	Take	0.0842	Pay	0.0994
Probably	0.0819	Understand	0.0825	Try	0.0994
AGI	0.0769	Computer	0.0798	Go	0.0941
LLM	0.0739	Maybe	0.0764	Big	0.0877
Drug	0.0735	Think	0.0748	Live	0.0862
Machine	0.0725	Day	0.0742	Sure	0.0853
Hope	0.0702	Read	0.0731	Real	0.0809
Robot	0.0702	Need	0.0726	Tell	0.0797
Good	0.0691	Thank	0.0715	Government	0.0779

In neutral discussions, posts convey ambiguous information. As shown in Figure 10B and Table 5, terms such as “change,” “challenge,” and “different” indicate that individuals are hesitant about the application of AI in medicine, expressing no clear preference for any single viewpoint. Although AI is recognized for its potential to improve healthcare services, its current implementation is not yet widespread and is perceived to require further testing over time.

In negative discussions, as illustrated in Figure 10C and Table 5, terms related to technological immaturity were prominent. High-frequency words in negative comments, such as “bad,” “control,” “argument,” and “problem,” reflect anxieties regarding errors, issues, or defects in medical AI. Users believe that AI technology is not yet mature and still faces many technical flaws, which could potentially undermine patient autonomy in medical decision-making and infringe upon individual health interests. Additionally, terms such as “company,” “money,” and “pay” suggest that concerns over the commercialization risks of medical AI also drive negative attitudes; there is a fear that AI-related corporations may prioritize profit over patient health, thereby neglecting medical ethics and equity. Negative comments featuring “job” and “human” reflect a tendency to compare AI with humans and a fear that AI will replace human physicians.

Notably, while “job” and “work” both refer to employment, these two terms exhibit distinct semantic divergence across different contexts. Specifically, the term “job” appears most frequently within the negative sentiment category (TF-IDF score: 0.1935) and is often used to express unemployment anxiety or professional threats. For instance, a paraphrased negative comment captures this anxiety: “It’s not about the work itself, it’s about my job. If an AI can do 90% of what a radiologist does, hospitals will just hire fewer of us to save money.” Another comment is more direct: “Every time I read about a new diagnostic AI, I just see another threat to my future job security.” This pattern suggests that when users use the word “job,” the discussion often shifts from technical functionality to concerns about professional displacement. In contrast, “work” is more common in the neutral category (TF-IDF score: 0.1208), focusing more on general work processes without a distinct emotional bias. For example, one paraphrased comment states: “Using AI at work helps me finish tasks faster and stay organized, but it doesn’t really replace what I do.”

### 4.3 Sentiment trend analysis

**Figure 11:**
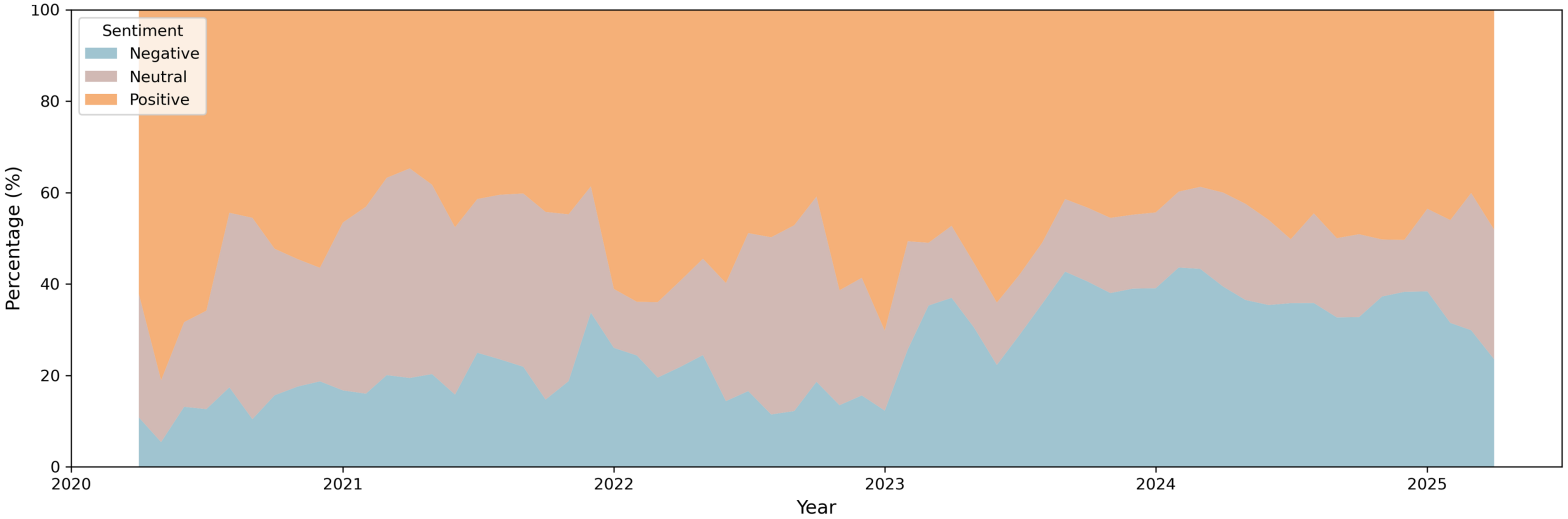
Sentiment trends from March 1, 2020, to March 31, 2025. This figure depicts the temporal trend of overall sentiment, with different colors representing distinct sentiment categories. The horizontal axis represents the timeline, while the vertical axis indicates the proportion, demonstrating the relative distribution of each sentiment category within the dataset across time.

We utilized a stacked area chart (Figure 11) to illustrate the temporal evolution of sentiment proportions regarding AI in healthcare among Reddit users from March 1, 2020, to March 31, 2025. This visualization effectively captures the shifting ratios of each sentiment category; however, fluctuations may be amplified during months with lower comment volumes. Therefore, the observed changes should be interpreted as representative trends rather than precise quantitative variances. In the figure, orange, gray, and blue represent the proportions of positive, neutral, and negative sentiments, respectively.

The results indicate that significant fluctuations in public sentiment primarily occurred after early 2023. Prior to 2023, the volume of positive posts and comments consistently outweighed negative entries, with neutral sentiments also being prevalent and negative sentiments relatively scarce, suggesting a generally optimistic outlook toward medical AI. However, starting in 2023, the volume of discussions surrounding medical AI on Reddit surged, with daily post counts during peak periods exceeding twice the previous baseline. During this time, the proportion of neutral entries decreased markedly, while negative entries rose significantly, progressively narrowing the gap between positive and negative sentiment shares. This suggests a shift toward more clearly valenced and more negative attitudes toward medical AI rather than definitive evidence of polarization.

These pronounced emotional shifts may be associated with the release and commercial availability of OpenAI’s multimodal model, GPT-4, in March 2023, but they should not be attributed to this event alone. Other concurrent factors, including the wider emergence of additional LLMs, public debates on AI regulation, and concerns about medical data security, may also have shaped public attitudes during this period. On one hand, GPT-4 accelerated the rapid application of medical AI, allowing the public to perceive its transition from conceptual frameworks to clinical implementation. On the other hand, the debut of GPT-4 triggered a competitive surge in large language models (LLMs), such as Google’s Med-PaLM, Anthropic’s Claude, and DeepSeek, which have been progressively integrated into the healthcare sector. These developments increased the public visibility of LLMs in medically relevant tasks and highlighted their potential in fields such as medical education and health advisory services (77). These developments may have made public concerns more concrete and contributed to more clearly expressed sentiment.

As an exploratory comparison, we examined sentiment changes around the broader generative AI diffusion period marked by the release of ChatGPT in November 2022 and GPT-4 in March 2023. Because the data are temporally ordered and may contain serial dependence, this comparison was interpreted cautiously as descriptive evidence rather than as a causal test. The results indicate an increase in the average monthly proportion of negative sentiment and a decrease in neutral sentiment after this diffusion period, suggesting that major generative AI milestones may coincide with changes in public emotion.

## 5 Discussion

### 5.1 Principal findings

With the rapid development of AI in healthcare, its potential risks and challenges have become increasingly apparent, leaving the public both filled with expectation and fraught with apprehension. The advancement of AI depends not only on technical progress but is also significantly influenced by public acceptance and trust. Public perceptions are often guided by how AI is portrayed in the media and encountered in daily life (78). Therefore, a profound understanding of these societal expectations is crucial for advancing technological applications and formulating policy. Currently, most research remains confined to interviews and questionnaires; although a few social media-based studies exist, their data and code are often not open-sourced, making independent verification and replication difficult and leaving a gap in comprehensive empirical research. To address this, our study uses Reddit data, collecting 1,020 posts and 36,555 comments from March 1, 2020, to March 31, 2025, to explore public focal points through BERTopic topic modeling and sentiment analysis.

We first compared our results with the prior work of Ocal (43), which concluded at the end of 2022. Our study covers the period of rapid public diffusion of generative AI. The comparison indicates that the structure and focus of current public discourse have shifted significantly. The intensity of discussion has surged, emotional attitudes have become more explicit, and core issues are more specific and realistic. The public focus has moved from the macro-potential of future AI toward concrete applications in real-world scenarios such as medical diagnosis and information management. Furthermore, to systematically interpret public attitudes, we employed the Technology Acceptance Model (TAM) (79) to investigate how perceived usefulness and perceived ease of use shape attitudes and behavioral intentions toward technology (80), subsequently analyzing the core factors influencing these perceptions. In applying TAM, positive discussions about assistance, efficiency, and diagnostic support were interpreted as expressions of perceived usefulness, whereas concerns about errors, privacy, commercialization, and physician displacement were interpreted as perceived risks that may weaken trust and acceptance.

To answer RQ1, results indicate that the most prominent topic is “AI and Human-Centered Healthcare” (71.54%), particularly regarding human labor (55.11%). This exhibits a clear “long-tail distribution” (81), reflecting structural public concerns about technology reshaping human roles in medical practice. In social media topic modeling, this characteristic is viewed as a natural phenomenon reflecting authentic public priorities rather than a data flaw requiring correction. These results suggest that Reddit users were less focused on abstract algorithmic performance, but rather with how technology alters the relationship between medical practice and human roles. Public attitudes are especially sensitive when AI intervenes in physician boundaries, decision-making authority, or patient communication. This concentrated focus reflects the tension between utility and social values: while people hope AI improves efficiency, they simultaneously fear the erosion of human care and professional autonomy. This finding aligns with previous research (9), suggesting that when evaluating emerging technologies, the public considers not only instrumental attributes but also ethical and identity-related factors. Although AI provides efficient solutions in healthcare, human interaction is still widely regarded as an irreplaceable value. Additionally, this study found that discussions on AI regarding public health monitoring and medical regulation accounted for 2.96 and 2.12%, respectively. This represents an increase compared to a previous study on Sina Weibo, which noted that only 0.5% of users focused on such issues (17). We speculate this difference stems from cultural backgrounds, institutional structures, and differing stages of technological awareness, as well as the higher proportion of tech-savvy and professional audiences on Reddit.

To answer RQ2, we found that while overall public sentiment is primarily positive (41.4%), the gap with negative sentiment (35.1%) is narrow, indicating a divided and increasingly cautious sentiment pattern. Positive sentiment stems largely from the recognition of AI’s usefulness, specifically expectations for improved diagnostic efficiency and clinical assistance. Conversely, significant negative sentiment is driven by external variables, particularly the perceived risk of misdiagnosis and privacy breaches, as well as distrust toward the corporate powers behind the technology. When AI demonstrates reliable performance and clear personal or societal benefits, its perceived usefulness increases, fostering positive attitudes. Conversely, if applications lack transparency, exhibit algorithmic bias, or trigger ethical controversy, the rise in perceived risk undermines the positive impacts of ease of use and utility, pushing the public toward caution or resistance. This changing sentiment pattern suggests that medical AI acceptance is not a static outcome but a dynamic process shaped by the interaction between perceived usefulness, perceived risk, and trust. Notably, the proportion of neutral sentiment in this study (23.8%) is significantly lower than the 65.7% (17) and 35% (15) reported in prior studies, indicating that as AI becomes more pervasive, public attitude is moving away from early ambiguous observation toward a clearer stance of support or skepticism.

To answer RQ3, the results suggest that fluctuations in public sentiment may be associated with major technological milestones. Taking the release of GPT-4 as an example, the event transformed AI from an abstract concept into a tangible tool. This not only enhanced the public’s intuitive understanding of usefulness and ease of use but also made risks, such as professional replacement and liability definition, more concrete, making related concerns more visible. Collectively, these phenomena reveal that public attitudes toward medical AI are not static but exist within an evolving dynamic system driven by key technical breakthroughs and policy governance.

Beyond these findings, the results of this study should be understood within the unique social and cultural context of Reddit. Reddit is not a neutral discursive space; its user structure and subreddit cultures, particularly in tech-oriented communities, have long reflected a leaning toward techno-optimism. This may partially explain the higher proportion of positive sentiment. However, more tellingly, even within this tech-friendly environment, users exhibit significant critical awareness and ethical sensitivity. The open and anonymous nature of the platform encourages users to directly express concerns regarding algorithmic bias and surveillance risks, illustrating that the social acceptance of technology involves public negotiation over trust, responsibility, and social values. Accordingly, claims in this study about public perception and trust should be understood as referring to English-language Reddit discourse rather than to the broader population as a whole. As an exploratory post-level check, we also compared the distribution of retained posts across major subreddit contexts. Health- and medical-technology-oriented communities, such as HealthIT, MedTech, HealthAI, HealthcareAI, and PublicHealth, showed relatively higher retention rates than broader general AI or technology communities. This pattern suggests that the filtering process preferentially retained discussions more directly related to medical AI, although it should not be interpreted as a full subreddit-stratified sentiment analysis.

### 5.2 Implications

Based on the research findings, several key implications can be derived:

1 Critical Technological Milestones as Focal Points for Public Attitude Guidance: The release of large-scale models, exemplified by GPT-4, coincided with increased discussion intensity and a more negative sentiment pattern. This indicates that major technological updates are not merely markers of progress but are also critical junctures for public risk perception and attitude formation. Policymakers, developers, and healthcare institutions should view such milestones as windows for proactive communication, guiding public opinion and building trust through authoritative information disclosure and by addressing core concerns.
2 Public Preference for Human–AI Collaboration over Technological Replacement: The study demonstrates that the public does not reject medical AI inherently; rather, they expect it to play a supportive role, allowing physicians to focus on tasks requiring higher-level judgment and humanistic care. Consequently, stakeholders should emphasize the supportive functions of AI for clinicians in product design and public messaging, avoiding narratives that reinforce the idea of “replacing doctors.”
3 Algorithmic Transparency as a Cornerstone for Building Public Trust: The public maintains a cautious stance regarding the commercial motives, opaque algorithms, and data utilization associated with medical AI. The long-term integration of medical AI necessitates the establishment of clear regulations concerning algorithmic principles, data usage, and the delineation of responsibility, alongside the implementation of feedback mechanisms to enhance system interpretability.
4 Focus on Practical Impacts Rather than Macro-Design: Results suggest that the public is more concerned with the actual effects of medical AI on the physician–patient relationship, privacy, and individual rights than with abstract technological visions. Future governance should be rooted in specific application scenarios, and should translate public concerns about dignity, privacy, and responsibility into technical design and regulatory practice.

### 5.3 Limitations

This study has the following limitations:

1 Data Source Singularity and Limited Cross-Platform Representativeness: The research relies exclusively on the Reddit platform, where 44.48% of the user base is from the United States (82). In addition, the English-language restriction may underrepresent non-English-speaking populations and regional perspectives on medical AI. This makes it difficult to fully represent the global public, meaning the conclusions are primarily applicable to the specific context of the English-speaking Reddit community. Furthermore, by not including video platforms such as YouTube, the study struggles to capture multimodal interactions and sentiment polarization driven by algorithmic recommendation mechanisms. Future research should employ cross-platform comparisons and multimodal analysis to overcome the limitations of a single-text platform in constructing a comprehensive discourse landscape.
2 Absence of Sociodemographic Characteristics Restricting Analytical Depth: Due to Reddit’s privacy protection measures, this study could not obtain demographic information such as user age, occupation, or nationality. This restricted the systematic comparison of attitudinal differences toward medical AI across various social groups and, to some extent, weakened the in-depth analysis of the structural characteristics of public acceptance. Future research could integrate survey data or sociodemographic proxy variables to perform a more granular analysis of public cognitive structures.
3 Temporal Constraints and Incomplete Data Sampling: Internet-based social cognition is characterized by dynamic evolution. This study focuses only on the specific period from March 1, 2020, to March 31, 2025, which may fail to capture background information from the early development of medical AI or long-term trends in public opinion evolution. Simultaneously, due to retrieval technology constraints, not all relevant discussions were included in the analysis, limiting our ability to comprehensively evaluate public perceptions. Future research should characterize the dynamic changes in public cognition more systematically by extending the time span and optimizing data acquisition strategies.

## 6 Conclusion

This study analyzed public perceptions of medical AI on the Reddit platform, including predominant topics, sentimental patterns, and emotional trends. The results indicate that the public generally maintains a positive outlook, focusing primarily on the development of medical AI and its impact on human roles and work practices within healthcare services. Furthermore, shifts in public sentiment may be associated with major AI-related events and show a shift toward more clearly valenced and more negative attitudes. To better promote the sustainable development of medical artificial intelligence, future efforts should move beyond technical optimization. Technology developers, medical institutions, and policymakers need to communicate the supportive role of AI in human–AI collaboration and establish mechanisms for transparency and accountability. As suggested by the Reddit discussions analyzed in this study, building public trust is not only a technical challenge but also a social and communicative one.

## Data Availability

The datasets presented in this study can be found in online repositories. The names of the repository/repositories and accession number(s) can be found in the article/Supplementary material.
