# Supplementary material for: Public perceptions of AI in healthcare: a large-scale BERTopic and sentiment analysis of Reddit discussions
Source: Front Public Health. 2026 Jun 2;14:1839898. doi: 10.3389/fpubh.2026.1839898 (PMC13269206; doi:10.3389/fpubh.2026.1839898)
Supplement: Supplementary file 1 [file Data_Sheet_1.pdf]

Retrieval Keyword List

The Reddit retrieval strategy used AI-related terms and healthcare-related terms. The main retrieval logic combined these two groups using Boolean operators.

AI-related terms

AI, Artificial Intelligence, Machine Learning, ML, Deep Learning, DL, NLP, Natural Language Processing, Computer Vision

Healthcare-related terms

healthcare, medical, medicine, clinical, patient, diagnostic, diagnosis, treatment, therapy, hospital, health, pharma, pharmaceutical, drug, radiology, pathology, genomics, digital health, healthtech, medtech

The Boolean retrieval structure was:

```
(AI OR "Artificial Intelligence" OR "Machine Learning" OR ML OR "Deep Learning" OR DL OR NLP OR "Natural Language Processing" OR "Computer Vision")
AND
(healthcare OR medical OR medicine OR clinical OR patient OR diagnostic OR diagnosis OR treatment OR therapy OR hospital OR health OR pharma OR pharmaceutical OR drug OR radiology OR pathology OR genomics OR "digital health" OR healthtech OR medtech)
```

In the implementation, title-level retrieval also used:

```
title: (AI OR "Artificial Intelligence") AND (health OR medical)
```

Additional query phrases considered in the retrieval script included:

```
Medical AI, Healthcare AI, Artificial Intelligence in Healthcare, AI in Medicine, AI in Healthcare, AI Diagnosis, Medical Technology AI, AI in Hospitals, AI for Doctors, AI for Patients, AI Diagnosis Tools, AI Surgery Assistance, AI Medical Imaging, AI Cancer Detection, AI in Radiology, AI in Pathology, AI in Drug Discovery, AI in Mental Health, ChatGPT for Healthcare, AI powered Medical Devices, Trust in Medical AI, Ethics of AI in Healthcare, AI and Patient Privacy, AI replacing Doctors, Fear of AI in Medicine, AI in Healthcare Opinions, AI Bias in Medicine, AI and Medical Errors, AI and Patient Safety, Doctors vs AI, AI and Medical Careers, AI and Nurses, AI Impact on Healthcare Jobs, Future of Medicine with AI, GPT4 in Medicine, Chatbots in Healthcare, AI powered Healthcare Apps, AI for Telemedicine, AI vs Human Doctors, Patient Perspective on AI, Doctors on AI, Public Opinion on AI in Healthcare, AI in Medicine Pros and Cons
```

Cleaning Keyword List

The cleaning process used relevance filters and noise filters. A post was retained only when it contained both AI-related and healthcare-related relevance terms and did not meet the spam/noise exclusion criteria.

AI-related relevance filters

AI, Artificial Intelligence, GPT, LLM, Assistance, ChatGPT, GPT-4, AI-powered, Chatbots

Healthcare-related relevance filters

Medical, Healthcare, Pathology, Diagnosis, Doctors, Patients, Cancer, Radiology, AI

Noise and spam exclusion filters

ad, ads, advertisement, promo, promotion, buy, sell, offer, sale, discount, deal, clearance, cheap, free, giveaway, bargain, coupon, limited time, special offer, scam, fraud, investment, get rich, quick money, make money, easy cash, earn, profit, passive income, pyramid scheme, binary option, forex, crypto, bitcoin, ethereum, nft, click here, visit, link, subscribe, follow, check out, http, https, www, signup, register, join now, don't miss, exclusive, limited, access now, download, watch now, buy now, order now, reserve your spot, act now, onlyfans, porn, xxx, nsfw, adult, dating, nude, explicit, sex, escort, sugar daddy, camgirl, hot singles, live chat, sexting, fetish, webcam, milf, bdsm, 18+, premium content, casino, poker, bet, lottery, jackpot, win big, gambling, slots, wager, bingo, sports betting, roulette, blackjack, scratch card, free spin, fake, replica, knockoff, counterfeit, cheap version, bootleg, copy, scammer, imitation, low-quality, phishing, miracle, magic, weight loss, diet pill, fat burner, cure, detox, anti-aging, supplement, boost, enhance, no side effects, natural remedy, herbal, guaranteed results, instant relief, health scam, free game, game cheat, hack, mod, cheat code, unlimited, unlock, skins, aimbot, wallhack, free gems, game booster, email me, contact us, phone number, dm me, message me, inbox me, reach out, unbelievable, amazing, incredible, shocking, breaking news, viral, must see, trending, life-changing, you won't believe, never seen before, survey, poll, pay-to-win, cash app, venmo, paypal, zelle, gift card, voucher, rebate, rewards, free trial, sign up, join us, membership, vip, job opportunity, work from home, remote job, freelance, part-time job, easy job, no experience needed, start today, be your own boss, startup kit, earn online, career opportunity, training, online course, certification, spam, junk, hoax, viral content, fake news, chain message, forward this, share now, copy-paste, spread the word, unverified

The cleaning script also excluded posts with excessive links. Specifically, posts were treated as spam if the title contained more than two URLs or the body contained more than one URL. Extremely long posts with body text of 1,500 characters or more were also excluded during relevance filtering.

System Prompt

```
#### Role
- Name: AI Healthcare Sentiment Analysis Expert
- Task: Analyze social media sentiment regarding AI in Healthcare.

#### Capabilities
- Relevance: Identify content directly related to AI in medical contexts.
- Context: Analyze relationships between posts and comments.
- Classification: Accurately determine author's sentiment.

#### Sentiment Criteria
- Positive: Benefits, progress, or success stories.
- Neutral: Factual statements without clear bias.
- Negative: Risks, ethical concerns, or failures.
- Meaningless: Irrelevant content (ads, games, etc.).

#### Output Instructions
- Input: Social media text (post or comment).
- Output: JSON format.
  - sentiment: (Positive/Neutral/Negative)
  - topic: One word (Application, Privacy, Patient-Doctor, Medicine, Employment, Safety, Newcomer, Ethics, Future, Fairness, Education, Cost, Bias, Mental)
  - relevance: Integer between 0 and 10.

#### Special Notes
- For comments, evaluate based on the thread context.
- relevance: 0 for irrelevant/ads, 10 for highly focused medical AI discussion.

EXAMPLE JSON OUTPUT:
{
    "results": [
        {
            "id": "1awin1",
            "sentiment": "Positive",
            "topic": "Application",
            "relevance": 8
        },
        {
            "id": "1bekw2",
            "sentiment": "Negative",
            "topic": "Privacy",
            "relevance": 2
        }
    ]
}
```

User Prompt Template

```
id: {id}
Type: {type}
Post Title: {title}
Post Body: {selftext}
Parent Comment: {parent_body}
Comment Body: {body}
```

Where:

- {id}: unique Reddit post or comment identifier.
- {type}: post or comment.
- {title}: title of the Reddit post.
- {selftext}: body text of the Reddit post.
- {parent_body}: parent comment text for nested comments; "None" for posts or top-level comments.
- {body}: comment body; "None" for post-level classification.
